# Healthy diets can create environmental trade-offs, depending on how diet quality is measured

**DOI:** 10.1186/s12937-020-00629-6

**Published:** 2020-10-27

**Authors:** Zach Conrad, Nicole Tichenor Blackstone, Eric D. Roy

**Affiliations:** 1 Department of Health Sciences, William & Mary, Williamsburg, VA 23185 USA; 2 Division of Agriculture, Food, and Environment, Friedman School of Nutrition Science and Policy, Tufts University, Boston, MA 02111 USA; 3 Rubenstein School of Environment and Natural Resources, University of Vermont, 81 Carrigan Dr, Burlington, VT 05405 USA; 4 Gund Institute for Environment, University of Vermont, 210 Colchester Ave, Burlington, VT 05405 USA

**Keywords:** Diet quality, Sustainability, HEI, AHEI, NHANES, Waste

## Abstract

**Background:**

There is an urgent need to assess the linkages between diet patterns and environmental sustainability in order to meet global targets for reducing premature mortality and improving sustainable management of natural resources. This study fills an important research gap by evaluating the relationship between incremental differences in diet quality and multiple environmental burdens, while also accounting for the separate contributions of retail losses, inedible portions, and consumer waste.

**Methods:**

Cross sectional, nationally-representative data on food intake in the United States were acquired from the National Health and Nutrition Examination Survey (2005–2016), and were linked with nationally-representative data on food loss and waste from published literature. Survey-weighted procedures estimated daily per capita food retail loss, food waste, inedible portions, and consumed food, and were summed to represent Total Food Demand. Diet quality was measured using the Healthy Eating Index-2015 and the Alternative Healthy Eating Index-2010. Data on food intake, loss, and waste were inputted into the US Foodprint Model to estimate the amount of agricultural land, fertilizer nutrients, pesticides, and irrigation water used to produce food.

**Results:**

This study included dietary data from 50,014 individuals aged ≥2 y. Higher diet quality (HEI-2015 and AHEI-2010) was associated with greater per capita Total Food Demand, as well as greater retail loss, inedible portions, consumer waste, and consumed food (*P* < 0.001 for all comparisons). Consumed food accounted for 56–74% of agricultural resource use (land, fertilizer nutrients, pesticides, and irrigation water), retail loss accounted for 4–6%, inedible portions accounted for 2–15%, and consumer waste accounted for 20–23%. Higher diet quality was associated with lower use of agricultural land, but the relationship to other agricultural resources was dependent on the tool used to measure diet quality (HEI-2015 vs. AHEI-2010).

**Conclusions:**

Over one-quarter of the agricultural inputs used to produce Total Food Demand were attributable to edible food that was not consumed. Importantly, this study also demonstrates that the relationship between diet quality and environmental sustainability depends on how diet quality is measured. These findings have implications for the development of sustainable dietary guidelines, which requires balancing population-level nutritional needs with the environmental impacts of food choices.

## Background

The global imperative to improve diet quality while simultaneously reducing environmental burdens is one of society’s most pressing challenges today. Suboptimal diet quality is now the leading behavioral risk factor for premature death [1], accounting for over 11 million deaths worldwide [2]. Diets high in sodium, and low in whole grains, fruits, vegetables, and nuts and seeds account for a substantial share of this burden due to the detrimental effects on vascular integrity, metabolic regulation, and gastrointestinal health [2]. These conditions play a major role in the etiology of coronary heart disease, stroke, diabetes, and neoplasms, which cumulatively represent the predominant share of premature death and disability worldwide [2]. According to recent estimates, improved diet quality could avert nearly 25% of premature deaths globally [3]. Meanwhile, massive amounts of agricultural resources are mobilized throughout the world to support current diet patterns, including non-renewable energy, fertilizer nutrients, pesticides, agricultural land, and water; all of which are linked with substantial environmental burdens, including greenhouse gas emissions, eutrophication, acidification, biodiversity loss, soil erosion, and water scarcity [4, 5].

In recognition of these dual health and environmental threats, the United Nations Sustainable Development Goals set ambitious targets for 2030. These include a reduction of premature mortality from non-communicable diseases (target 3.4) and sustainable management of natural resources (target 12.2) [6]. Clearly, there is an urgent need to assess the linkages between diet patterns, health outcomes, and environmental sustainability, and research has expanded greatly in this domain [7], with several high-profile studies published recently [5, 8–12].

However, there are several notable gaps in the literature that preclude a more complete understanding of the linkage between diet patterns, health outcomes, and environmental sustainability. In most cases, studies have assessed theoretical diet patterns that reflected perfect adherence to dietary recommendations [9–11, 13–16] or varying amounts of animal protein [4, 10, 15, 17, 18], rather than self-selected diets [19–22] that vary by validated measures of diet quality [23, 24]. The literature is also replete with assessments of greenhouse gas emissions at the expense of other environmental sustainability indicators [7, 25, 26]. To the best of our knowledge, no studies have evaluated the relationship between incremental differences in diet quality and multiple environmental burdens, while also accounting for the separate contributions of retail losses, inedible portions, and consumer waste.

To fill these important research gaps, the present study assesses the relationship between observed diet quality among a nationally-representative sample of over 50 thousand Americans and the amount of agricultural land, fertilizer nutrients, pesticides, and irrigation water used to produce food. We focus on the United States (US) because suboptimal diet quality is the leading risk factor for premature death and a predominant risk factor for morbidity [27], and the majority of consumed food is produced domestically [28, 29]. Thus, shifts in diet quality among Americans would have meaningful implications for environmental sustainability within US borders and beyond.

## Methods

### Dietary data

Data on daily food intake at the individual level were acquired from the National Health and Nutrition Examination Survey (NHANES) 2005–2016 [30]. NHANES is a continuous, cross-sectional, multi-stage survey maintained by the National Center for Health Statistics (NCHS). Approximately 5000 individuals participate in the survey annually, and data are released on a two-year cycle. Each individual completes a 24-h recall administered by a trained interviewer using United States Department of Agriculture’s (USDA) Automated Multiple Pass Method [31], and a subset of the study population completes a subsequent 24-h recall by telephone on a non-consecutive day. Data from day 1 were used because this represents per capita intake [32]. NHANES provides dietary data as reported by individuals, which often include mixed dishes composed of multiple foods, such as lasagna. Disaggregation of these mixed dishes into component foods was performed with the Food Commodity Intake Database (FCID) [33], which was developed by the US Environmental Protection Agency (US EPA). FCID (2005–2010) provides data on the amount of nearly 500 foods included in each dish listed in NHANES in their as-consumed forms. FCID has not been updated since 2010 to link with the new food codes added to NHANES since then, so the loss and waste rates of these foods were not estimated.

### Food loss and waste

Each food in FCID was linked with a distinct food commodity (i.e., ingredient) from USDA Loss-adjusted Food Availability data series (LAFA), which provides estimates of retail loss, inedible portions, and consumer waste (i.e., loss/waste categories) for over 200 commodities (Supplemental Figure 1) [34]. Retail loss represents food that was discarded in supermarkets, convenience stores, and other food retail outlets (except restaurants and other outlets that serve primarily prepared foods) due to spoilage, damage, blemishes, moisture loss, overstocking, or any other reason. Inedible portions include pits, cores, and some seeds and peels that are discarded at the consumer level, and consumer waste includes edible portions of food that are discarded for any reason, including spoilage, blemishes, spillage, distaste for leftover food, and lack of knowledge about food selection strategies, food preparation, or storage options. The details of this procedure are described elsewhere [35, 36] and depicted in Supplemental Figure 1.

### Diet quality assessment

Multiple, validated instruments were used to provide a robust assessment of diet quality. The Healthy Eating Index (HEI-2015) [37, 38] was designed to evaluate compliance with the 2015–2020 Dietary Guidelines for Americans [39], and the Alternative Healthy Eating Index (AHEI-2010) [40] was constructed based on foods and nutrients associated with chronic disease risk [40]. Both instruments are discussed below.

HEI-2015 includes 13 components (total fruit, whole fruit, total vegetables, greens and beans, whole grains, dairy, total protein foods, seafood and plant proteins, unsaturated:saturated fats, refined grains, sodium, added sugars, and saturated fats), and the consumption amounts for each component are standardized to a 1000 kcal basis (Supplemental Table 1). Each component has different scoring standards that range from 0 to 5 or 0–10, with greater scores being awarded for greater consumption of total fruit, whole fruit, total vegetables, greens and beans, whole grains, dairy, total protein foods, seafood and plant proteins, and unsaturated:saturated fats. Intake of refined grains, sodium, added sugars, and saturated fats are reverse scored so that greater scores are awarded for lesser consumption. For each individual providing dietary data in NHANES, the component scores were summed to compute an overall score with a maximum of 100. Mean scores were appropriately computed using the population-ratio method [41].

This study used 10 out of the 11 components of the original AHEI-2010: vegetables, fruit, whole grains, nuts and legumes, long-chain ω-3 fats, total polyunsaturated fats, sugar-sweetened beverages and fruit juice, red and processed meat, sodium, and alcohol (Supplemental Table 1). *Trans* fats were not included in this study because NHANES does not provide complete data on *trans* fat content of foods, and population-level intake in the US has decreased markedly since 1999 [42]. Each component is scored on a scale of 0 to 10, and each has its own standards for the amount of points awarded for consumption amount. Intake of vegetables, fruit, whole grains, nuts and legumes, long-chain ω-3 fats, and total polyunsaturated fats are scored such that greater consumption is awarded greater points. Sugar sweetened beverages and fruit juice, red and processed meat, and sodium are reverse scored so that greater scores are awarded for lesser consumption, and greater scores are awarded for moderate consumption of alcohol. For each individual providing dietary data in NHANES, the component scores were summed to compute an overall score with a maximum of 100. AHEI-2010 was originally developed using a source population of adults and was not energy adjusted [40], so we made several modifications to adapt this instrument to a population that includes individuals < 18 y. Consumption amounts for all individuals were energy adjusted to the mean energy intake of the source population (1849 kcal/d) [40], which has precedent [43]; and individuals < 18 y were awarded 10 points for the alcohol component if they reported zero consumption and were awarded zero points if they reported any alcohol consumption. All individuals were grouped by quintile of HEI-2015 and AHEI-2010 score, where quintile 1 represents the lowest diet quality and quintile 5 represents the highest diet quality.

### Modeling structure

The amount of agricultural land, fertilizer nutrients, pesticides, and irrigation water used to produce food was estimated using the US Foodprint Model, a biophysical simulation model that represents the US as a closed food system (Supplemental Figure 2) [44]. The US Foodprint Model accepts user-inputted data on daily per capita intake of 22 distinct food groups (grains; dark green vegetables; red and orange vegetables; dry beans, lentils, and peas; starchy vegetables; other vegetables; fruit; fluid milk and yogurt; cheese and other dairy; soy milk; nuts; tofu; beef; pork; chicken; turkey; eggs; seafood; plant oils; dairy fats; lard and tallow; and sweeteners), and embedded computations convert these as-consumed foods back to raw agricultural crops (grains, fruits, vegetables, legumes, nuts, sweeteners, feed grains and oilseeds, hay, cropland pasture, and permanent pasture) and the amount of agricultural land needed to produce those crops, by modeling the stepwise transformation of these foods as they move through the US food system. Key transformation parameters include population size, food processing conversions, livestock feed requirements, crop and livestock yields, availability of agricultural land, and suitability of agricultural land for food production. Additional computations account for multi-use crops (i.e., crops that are used to produce multiple products from equivalent mass) and multi-use cropland (cropland used to produce multiple crops during different parts of the year). Additional details are available elsewhere [44].

The original US Foodprint Model [44] was modified in the present study to enhance the robustness of model outputs. The embedded computations that account for loss and waste at the retail and consumer levels were nullified to avoid double counting, because the amount of food lost and wasted was inputted into the model separately to quantify their explicit association with the use of agricultural resources (embedded computations that account for loss and waste at the pre-retail stage of the food system were not nullified). Nationally-representative application rates (annual amount applied per land area) of fertilizer nutrients (nitrogen, phosphorus-P_2_O_5_, and potash-K_2_O), pesticides, and irrigation water were embedded into the model based on data acquired from USDA Agricultural Surveys (2002–2016) [45] and Farm and Ranch Irrigation Surveys (2003–2013) [46, 47]. Data on chemical use for hay and pasture are not available in USDA Agricultural Surveys; these data were estimated based on published literature and recommendations from agricultural Extension Service agents in top producing states [48–54]. Data on crop yields (mean of 2011–2015 for most crops) [45] and population size (2015) [55] were updated. Finally, a Monte Carlo simulation procedure with random, non-replacement draws was incorporated into the US Foodprint model to produce reliable estimates of population-level variation based on inter-individual variability of food intake from NHANES.

### Sensitivity analyses

Several sensitivity analyses were conducted to examine sources of uncertainty. FCID was used to disaggregate NHANES foods into their component ingredients, but this database has not been updated since 2010 and may not be applicable to subsequent NHANES surveys. To investigate whether FCID produced biased estimates of food intake over time, analyses were conducted for two distinct time points (2005–2016 and 2005–2010). Mean differences were estimated in grams and as proportions of total daily per capita demand overall and for each quintile. The AHEI-2010 scoring algorithm was modified to adapt it to a population that includes individuals < 18 y; so to examine whether this approach produced biased estimates of diet quality, analyses were conducted using both approaches (original and modified) for each age group (2–17 y and ≥ 18 y), and were tested against each other using Wald tests with *P* < 0.05.

### Main analyses

The per capita amount (grams) of food retail loss, inedible portions, consumer waste, and consumed food were estimated separately, and were summed to estimate Total Food Demand for each food group. The relationship between the amount of Total Food Demand (by loss/waste category and food group) and quintiles of diet quality (HEI-2015 and AHEI-2010) was assessed using simple linear regression models to test for trend, and additional models were adjusted for age (continuous) and sex (male/female). Diet quality estimates were energy adjusted using the density method, where food intake was standardized per 1000 kcal (HEI-2015) or per 1849 kcal (AHEI-2010), as discussed above. Standardized procedures and variables provided by NCHS [56] were used to account for the multistage probability sampling design of NHANES. The relationship between the amount of agricultural resources (agricultural land, fertilizer nutrients, pesticides, and irrigation water) used to produce Total Food Demand and quintiles of diet quality (HEI-2015 and AHEI-2010) was assessed using simple linear regression models to test for trend. Statistical significance was set at *P* < 0.05 for all assessments. SAS 9.4 (SAS Institute; Cary, NC) was used to estimate population-ratio HEI-2015 scores using the modified code and macros provided by the National Cancer Institute (discussed above) [57]. Stata16 (StataCorp; College Station, TX) was used for data management and all other analyses.

## Results

**Fig. 1:**
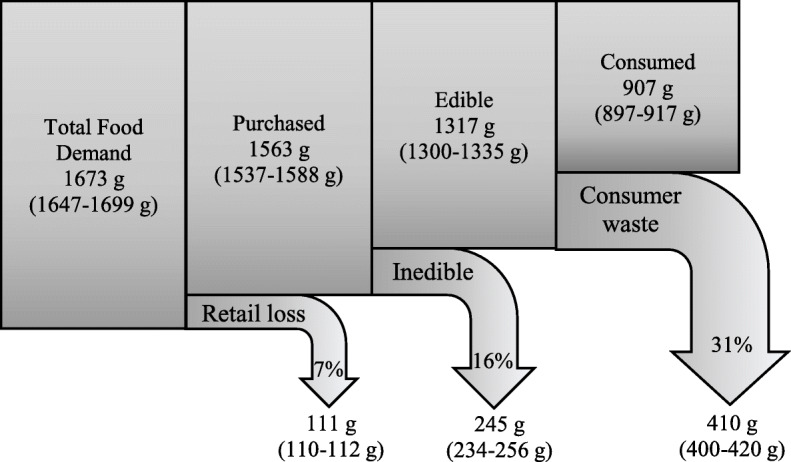
Daily per capita Total Food Demand, 2005–2016 (*n* = 50,014)

This study included 50,014 individuals ≥2 y who provided complete and reliable dietary data as determined by a trained NCHS interviewer (Supplemental Table 2). The mean overall HEI-2015 score was 58.4 (95% CI 57.7–59.0) out of 100 (Supplemental Table 3), and the mean overall AHEI-2010 score was 41.8 (41.4–42.2) out of 100 (Supplemental Table 4). Per capita Total Food Demand represented 1673 g (95% CI 1647–1699 g), and 7% (111 g, 110–112 g) was lost at the retail level (Fig. 1). Purchased food represented 1563 g (1537–1588 g), 16% (245 g, 234–256 g) of which was inedible. Of the remaining 1317 g (1300–1335 g) of edible food, 31% (410 g, 400–420 g) was wasted, and 907 g (897–917 g) was consumed.

**Fig. 2:**
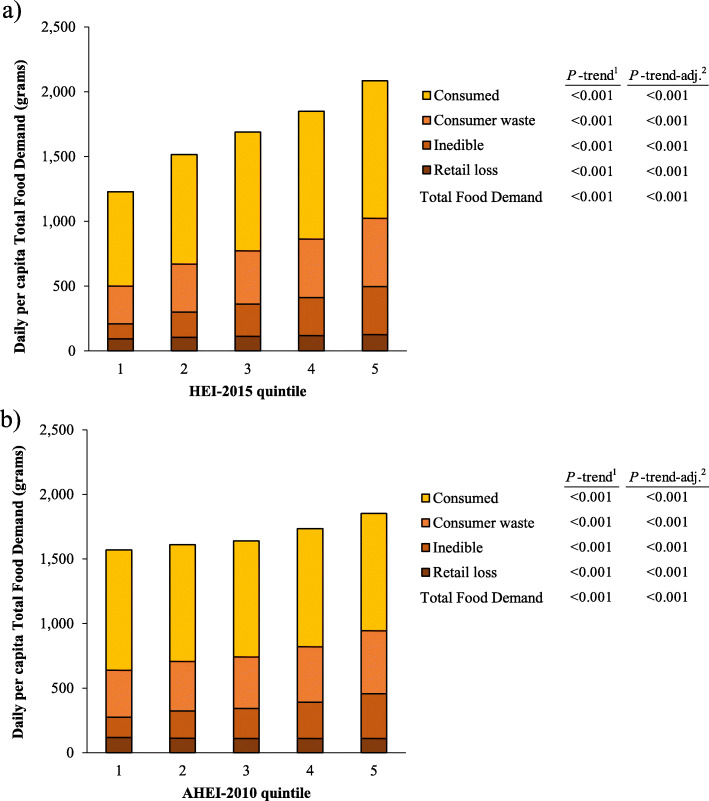
Daily per capita Total Food Demand (2005–2016) by **a**) Healthy Eating Index-2015 quintile, and **b**) Alternative Healthy Eating Index-2010 quintile (*n* = 50,014). Total Food Demand includes retail loss, inedible, consumer waste, and consumption. HEI-2015, Healthy Eating Index-2015. AHEI-2010, Alternative Healthy Eating Index-2010. Higher quintiles indicate higher diet quality. ^1^Test for linear trend across quintiles 1 through 5, not adjusted for covariates. ^2^Test for linear trend across quintiles 1 through 5, adjusted for age (continuous) and sex (male/female)

Higher diet quality (HEI-2015 and AHEI-2010) was associated with greater per capita Total Food Demand, as well as greater retail loss, inedible portions, consumer waste, and consumed food (*P* < 0.001 for all comparisons; Fig. 2). By food group, higher HEI-2015 scores were associated with greater Total Food Demand for dairy, soup, nuts and seeds, fruits and vegetables, table oils and salad dressing, and salty snacks (*P* < 0.001 for all comparisons; Supplemental Table 5). Higher AHEI-2010 scores were associated with greater Total Food Demand for soup, grains, nuts and seeds, fruits and vegetables, table oils and salad dressing, and salty snacks (*P* < 0.01 for all comparisons; Supplemental Table 6).

**Fig. 3:**
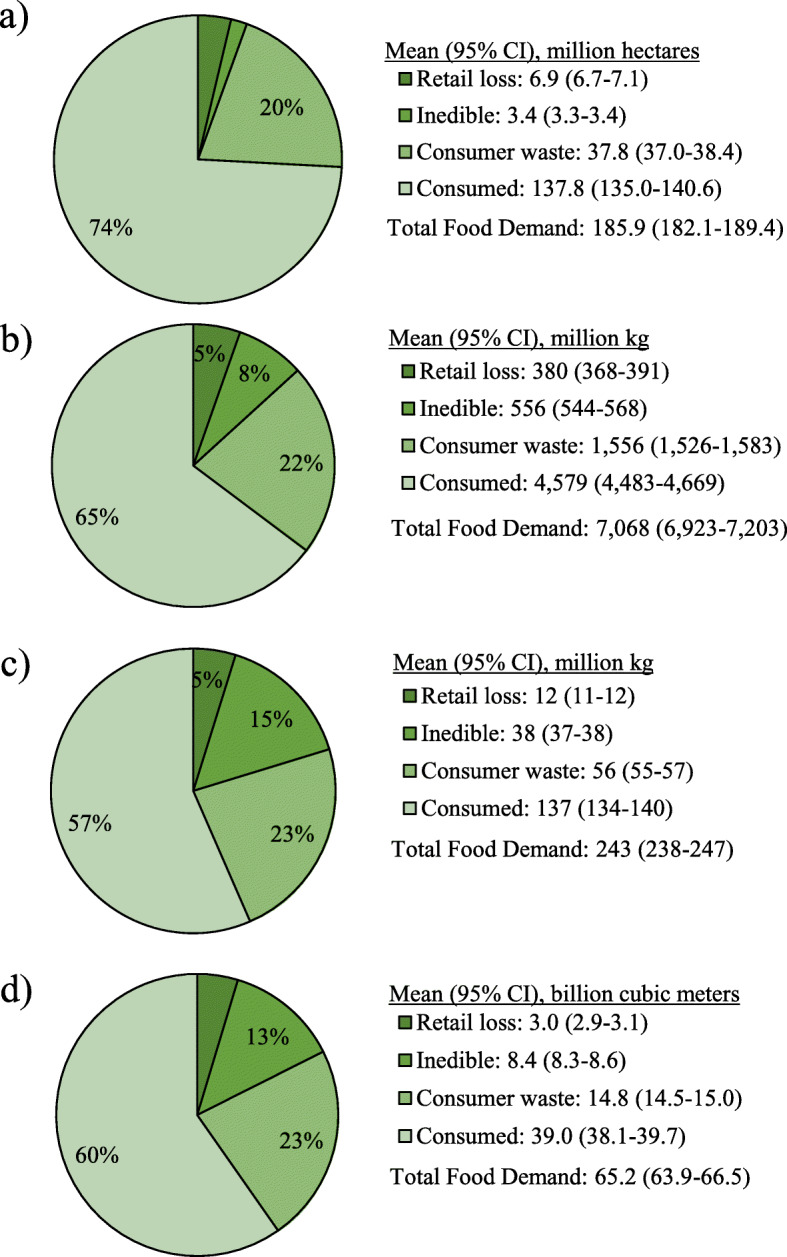
Annual amount of agricultural resources used to produce Total Food Demand: **a**) agricultural land, **b**) fertilizer nutrients, **c**) pesticides, and **d**) irrigation water. Total Food Demand includes retail loss, inedible, consumer waste, and consumption. Agricultural land includes, grains, fruits, vegetables, legumes, nuts, sweeteners, feed grains and oilseeds, hay, permanent pasture, and cropland pasture. Pesticides represent the sum of herbicides, insecticides, and fungicides. Fertilizer nutrients represent the sum of nitrogen, phosphorus (P_2_O_5_), and potash (K_2_O). Values < 5% are not labeled

The equivalent of 185.9 million hectares (95% CI 182.1–189.4 million hectares) of agricultural land, 7068 million kg (6923–7203 million kg) of fertilizer nutrients (N + P_2_O_5_ + K_2_O), 243 million kg (238–247 million kg) of pesticides, and 65.2 billion cubic meters (63.9–66.5 billion cubic meters) of irrigation water were used to produce Total Food Demand on an annual basis (Fig. 3). Consumed food accounted for 57–74% of each agricultural resource category, retail loss accounted for 4–5%, inedible portions accounted for 2–15%, and consumer waste accounted for 20–23%.

**Fig. 4:**
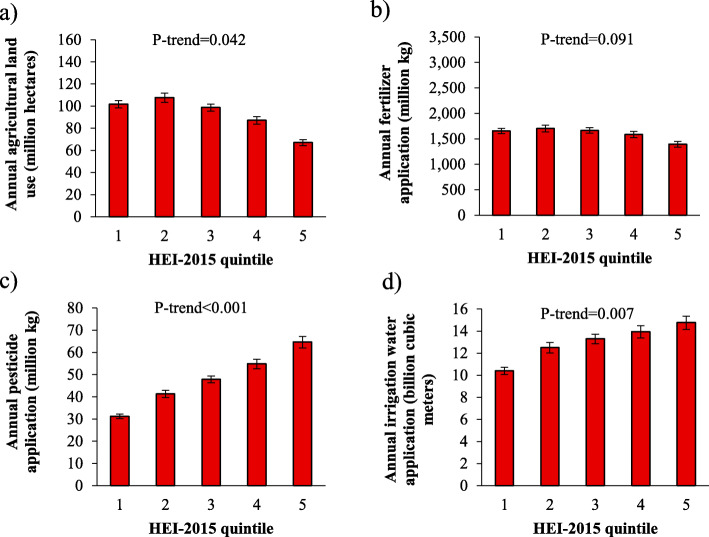
Annual amount of agricultural resources used to produce Total Food Demand, by Healthy Eating Index-2015 quintile: **a**) agricultural land, **b**) fertilizer nutrients, **c**) pesticides, and **d**) irrigation water. HEI-2015, Healthy Eating Index-2015. Higher quintiles represent higher diet quality. Total Food Demand includes retail loss, inedible, consumer waste, and consumption. Agricultural land includes, grains, fruits, vegetables, legumes, nuts, sweeteners, feed grains and oilseeds, hay, permanent pasture, and cropland pasture. Pesticides represent the sum of herbicides, insecticides, and fungicides. Fertilizer nutrients represent the sum of nitrogen, phosphorus (P_2_O_5_), and potash (K_2_O)

Relationships between diet quality and agricultural resource use were similar between HEI-2015 and AHEI-2010 for all land use categories except for total land (fertilizer nutrients, pesticides, and irrigation water), and feed grains and oilseeds (agricultural land, fertilizer nutrients, pesticides, and irrigation water; Table 1). Greater HEI-2015 scores (i.e. greater diet quality) were associated with less agricultural land use (*P* = 0.042), and greater use of pesticides (*P* < 0.001) and irrigation water (*P* = 0.007; Fig. 4). Across HEI-2015 quintiles (quintile 1 = lowest diet quality, quintile 5 = greatest diet quality), permanent pasture (*P* = 0.037) accounted for most of the decreased trend in agricultural land use (difference of 10.8 million hectares between quintiles 1 and 5; Supplemental Table 7). No difference (*P* = 0.091) across quintiles was observed for fertilizer nutrients (Supplemental Table 8), largely because the positive relationship between diet quality and use of fertilizer nutrients on land used to produce fruits and vegetables (increase of 121 million kg and 129 million kg, respectively, between quintiles 1 and 5) was compensated by a negative relationship between diet quality and fertilizer nutrients on land used to produce hay (decrease of 271 million kg between quintiles 1 and 5). Fruits accounted for most of the increased trend in pesticide use (*P* = 0.001; increase of 35 million kg between quintiles 1 and 5; Supplemental Table 9) and irrigation water use (*P* = 0.001; increase of 5452 million cubic meters between quintiles 1 and 5; Supplemental Table 10).

**Table 1:** Relationship between diet quality and agricultural resource use, by land use category

Land use category	Agricultural land	Fertilizer nutrients	Pesticides	Irrigation water
Total				
HEI-2015	↓	–	↑	↑
AHEI-2010	↓	↓	–	–
Grains				
HEI-2015	↓	↓	↓	↓
AHEI-2010	↓	↓	↓	↓
Fruits				
HEI-2015	↑	↑	↑	↑
AHEI-2010	↑	↑	↑	↑
Vegetables				
HEI-2015	↑	↑	↑	↑
AHEI-2010	↑	↑	↑	↑
Legumes				
HEI-2015	↑	↑	↑	↑
AHEI-2010	↑	↑	↑	↑
Nuts				
HEI-2015	↑	↑	↑	↑
AHEI-2010	↑	↑	↑	↑
Sweeteners				
HEI-2015	↓	↓	↓	↓
AHEI-2010	↓	↓	↓	↓
Feed grains and oilseeds				
HEI-2015	–	–	–	–
AHEI-2010	↓	↓	↓	↓
Hay				
HEI-2015	↓	↓	↓	↓
AHEI-2010	↓	↓	↓	↓
Cropland pasture				
HEI-2015	↓	–	–	↓
AHEI-2010	↓	–	–	↓
Permanent pasture				
HEI-2015	↓	–	–	↓
AHEI-2010	↓	–	–	↓

Total Food Demand represents the sum of retail waste, consumer waste, inedible portions, and consumed food

HEI-2015, Healthy Eating Index-2015

AHEI-2010, Alternative Healthy Eating Index-2010

Upward arrow (↑) represents a statistically significant (*P* < 0.05) positive relationship between diet quality and agricultural resource use, downward arrow (↓) represents a statistically significant negative relationship, and horizontal line (−) represents a non-statistically significant relationship

**Fig. 5:**
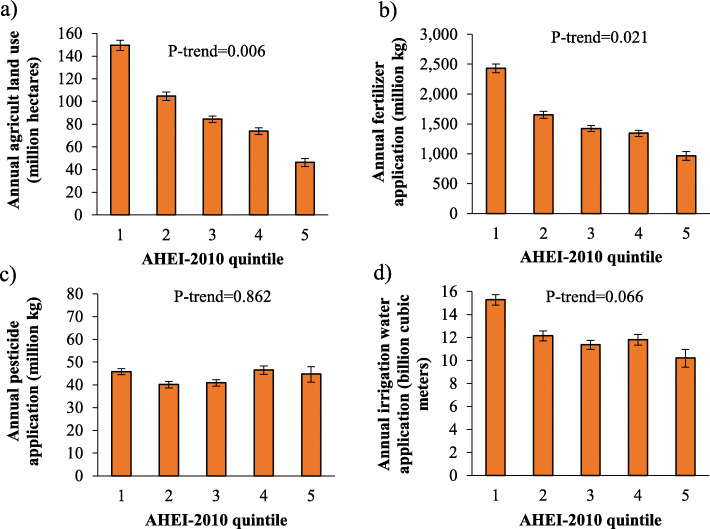
Annual amount of agricultural resources used to produce Total Food Demand, by Alternative Healthy Eating Index-2010 quintile: **a**) agricultural land use, **b**) irrigation water application, **c**) pesticide application, and **d**) fertilizer application. AHEI-2010, Alternative Healthy Eating Index-2010. Higher quintiles represent higher diet quality. Total Food Demand includes retail loss, inedible, consumer waste, and consumption. Agricultural land includes, grains, fruits, vegetables, legumes, nuts, sweeteners, feed grains and oilseeds, hay, permanent pasture, and cropland pasture. Pesticides represent the sum of herbicides, insecticides, and fungicides. Fertilizer nutrients represent the sum of nitrogen, phosphorus (P_2_O_5_), and potash (K_2_O)

Greater AHEI-2010 scores (i.e., greater diet quality) were associated with less agricultural land use (*P* = 0.006) and less use of fertilizer nutrients (*P* = 0.021; Fig. 5). Across AHEI-2010 quintiles (quintile 1 = lowest diet quality, quintile 5 = greatest diet quality), permanent pasture (*P* = 0.006) accounted for most of the decreased trend in agricultural land use (decrease of 31 million hectares between quintiles 1 and 5; Supplemental Table 11), and hay (*P* = 0.006) and feed grains and oilseeds (*P* = 0.004) accounted for most of the decreased trend in fertilizer nutrients (decrease of 731 million kg and 140 million kg, respectively, between quintiles 1 and 5; Supplemental Table 12). No difference (*P* = 0.862) across quintiles was observed for pesticides (Supplemental Table 13), largely because the positive relationship between diet quality and use of pesticides on land used to produce fruits and vegetables (increase between quintiles 1 and 5 of 8 million kg and 5 million kg, respectively) was compensated by a negative relationship between diet quality and use of pesticides on land used to produce hay and feed grains and oilseeds (decrease of 15 million kg and 3 million kg, respectively, between quintiles 1 and 5). No difference (*P* = 0.066) across quintiles was observed for irrigation water (Supplemental Table 14), largely because the positive relationship between diet quality and use of irrigation water on land used to produce fruits and vegetables (increase of 1243 million cubic meters and 1629 million cubic meters, respectively, between quintiles 1 and 5) was compensated by a negative relationship between diet quality and use of irrigation water on land used to produce hay (decrease of 4829 million cubic meters between quintiles 1 and 5).

Sensitivity analyses demonstrated that using FCID (2005–2010) to disaggregate NHANES foods (2005–2016) likely underestimated daily per capita Total Food Demand (Supplemental Table 15). When mean differences between the two time points were calculated as proportions of total food demand, there were no meaningful differences across diet quality quintiles. Separate sensitivity analyses demonstrated that the modified AHEI-2010 score was 0.390 points higher than the original score among individuals ≥18 y (*P* < 0.001), and no difference (*P* = 0.946) was observed among individuals < 18 y (Supplemental Table 16).

## Discussion

In this nationally-representative study of over 50 thousand Americans, we used an interdisciplinary modeling framework that integrated methods from nutritional epidemiology with food system science to evaluate the linkage between diet quality and environmental sustainability. Higher diet quality was associated with greater Total Food Demand, including greater retail loss, inedible portions, consumer waste, and consumed food. Higher diet quality was associated with lower use of agricultural land, but the relationship to fertilizer nutrients, pesticides, and irrigation water was dependent on the tool used to measure diet quality (HEI-2015 vs. AHEI-2010). Over one-quarter of the agricultural inputs used to produce Total Food Demand were attributable to edible food that was not consumed, including retail loss and consumer waste.

This study demonstrates that the relationship between diet quality and agricultural resource use depends on how diet quality is measured. Using AHEI-2010, we show that higher diet quality is associated with similar or decreased use of agricultural resources; whereas, when using HEI-2015, we show that higher diet quality is associated with tradeoffs: decreased land use, no difference in use of fertilizer nutrients, and greater use of pesticides and irrigation water. These mixed relationships reflect different approaches to measuring healthy diets, even though validation studies have shown that both indices adequately predict chronic disease risk in prospective cohorts [38, 40, 58–60]. HEI-2015 [37, 38] measures broad adherence to the Healthy Eating Patterns of the 2015–2020 Dietary Guidelines for Americans, which were derived using a food pattern modeling approach that optimized nutrient intake through consumption of a variety of foods within each food group [61]. In contrast, AHEI-2010 was constructed based on epidemiologic evidence that links specific foods and nutrients with chronic disease risk [40]. As a result, these indices use difference scoring standards, especially for fruit, meat, and dairy [38, 40], which largely explains their different associations with environmental sustainability in this study.

Compared to HEI-2015, AHEI-2010 uses more stringent scoring standards for fruit (fruit juice is scored as sugar sweetened beverage rather than a fruit), does not score dairy consumption (whereas HEI-2015 rewards greater consumption of dairy), and rewards lesser consumption of meat (whereas HEI-2015 does not score meat explicitly). Although HEI-2015 does reward greater intake of protein foods, which can indirectly reward greater meat and dairy intake among those that report consuming these foods, it also rewards lesser consumption saturated fat, which indirectly penalizes greater intake of animal-based foods (beef and dairy account of 15% and 18% of daily per capita saturated fat intake, respectively) [62]. As a result, the difference in consumption amounts between the lowest and highest AHEI-2010 quintiles in this study is + 322 g for fruit (+ 550 g using HEI-2015), − 121 g for meat (− 5 g using HEI-2015), and − 2 g for dairy (+ 106 g using HEI-2015). Consumer demand for these foods drives the amount of agricultural resources used to produce fruit, feed grains and oilseeds, and hay, which represent the predominant share of agricultural resources used to produce all food in this study. Although direction of the associations between diet quality and agricultural resource use were similar between HEI-2015 and AHEI-2010 within each land use category, the magnitude of these relationships were different (especially for fruit, meat, and dairy); which is apparent when the values for each land use category are summed to estimate results for total land area.

In a recent review, Reinhardt et al. (2020) [7] reported that a shift to the DGA recommended Healthy US-Style diet would result in similar or decreased agricultural land use, and similar or greater use of blue water, which is consistent with our findings. Few studies have evaluated the environmental impacts associated with incremental improvements in diet quality in the US using data collected directly from individuals, rather than theoretical diet patterns that reflect perfect adherence to national (or global) dietary recommendations. Others have observed that higher HEI-2015 scores were associated with less agricultural land use [63], greater food waste, and greater waste of pesticides and irrigation water [35]. Rose et al. demonstrated that individuals consuming diets with greater greenhouse gas emissions had lower HEI-2010 scores [23], although a modeling approach used by Hitaj et al. recently found that a shift from current consumption patterns to the DGA recommended diet pattern had a minimal effect (0.4% reduction) on greenhouse gas emissions without decreasing the intake of animal protein [22]. To the best of our knowledge, the present study is the first to evaluate the relationship between multiple indicators of environmental sustainability and incremental improvements in diet quality, using multiple measures of diet quality, and using dietary data collected directly from individuals.

The present study demonstrates that the relationship between diet quality and environmental sustainability is more nuanced than previously understood, and therefore challenges the notion that healthy diets are inherently more environmentally sustainable [64]. Conrad et al. demonstrated that healthier diets were associated with greater consumer food waste and associated agricultural resources [35], and Rose et al. demonstrated that diets responsible for greater greenhouse gas emissions had greater content of some micronutrients (vitamin A, vitamin D, choline, iron, calcium, and potassium) [23]. The present study also supports the main findings of several reviews that reported that healthier diets are not necessarily more environmentally sustainable [7, 65].

Fruits, vegetables, and nuts, which are key components of a healthy diet pattern, typically require substantially greater inputs per unit land area than most other foods [45]; and sugar-sweetened beverages and refined grains, which have been linked with detrimental health effects [66, 67], have relatively modest environmental impacts [5]. And although greater consumption of red and processed meat has been linked with meaningful health (and environmental) risks [7, 66, 67], the 2015–2020 Dietary Guidelines for Americans and corresponding diet quality index (HEI-2015) do not explicitly recommend limiting red and processed meat intake as part of a healthy diet. Diet quality indices vary in their measurement of these foods, which influences their associations with measures of environmental sustainability.

However, caution is warranted when ascribing a measure of environmental sustainability to any individual food product or group; what matters most is the balance of foods across an eating pattern. Thus, greater efforts are needed to assess the environmental impacts of individual, self-selected diet patterns rather than individual foods. Recently, the EAT-Lancet Commission on Healthy Diets from Sustainable Food Systems published a global recommended diet pattern to promote human health within environmental boundaries [8], and others have developed methods to convert these recommendations to a US population [68]. A logical next step for future research is to evaluate the environmental burden associated with the adoption of the EAT-Lancet diet pattern in the US.

Our findings have implications for the development of sustainable dietary guidelines in the US and beyond. First, the positive association between food loss/waste and diet quality suggests that policy aimed at improving the healthfulness of diets may need to be coupled with efforts to reduce food loss/waste to avoid unintended consequences of pursuing these aims independently. Precedent exists in the Dietary Guidelines for Americans (DGA) for providing consumer guidance beyond food choices; the DGA also include recommendations for physical activity and safe food handling [39]. Second, our finding that greater compliance with the DGA is associated with tradeoffs for agricultural resource use (lower land use but greater use of pesticides and irrigation water) illustrates that dietary recommendations may be moderated by additional considerations of environmental sustainability. In other words, the nutritionally-optimal intake of specific foods may not be optimal for the environment, or may only yield environmentally-positive impacts under certain circumstances. Human diets are significant contributors to ecological crises like climate change that require near-term, large-scale mitigation approaches [8]. As such, developing the institutional processes, political will, and interdisciplinary knowledge required to create sustainable dietary guidelines is a complex endeavor that requires balancing nutritional needs with environmental impact, but should nonetheless be high priority [69].

The strengths of this study include the assessment of individual, self-selected diet patterns differentiated by incremental differences in diet quality, rather than theoretical diets that reflect perfect adherence to dietary recommendations, which enhances the practical application of our findings [7]. The robustness of diet quality assessment was enhanced by using multiple, validated tools that consider all foods reportedly consumed, rather than utilizing a single measure of diet quality or focusing on individual food groups [70]. Diet quality assessment was also adjusted for energy intake to reduce the observed confounding effect of energy intake on food intake and sustainability outcomes [26, 70], and to adapt AHEI-2010 to a population that includes individuals < 18 y; sensitivity analyses demonstrated that this modification did not introduce any meaningful bias into the analyses. Finally, this study fills a gap in the literature by assessing multiple resource use indicators of environmental sustainability, which provides a comprehensive evaluation of the environmental impacts of diet patterns [25, 26].

The limitations of this study should also be considered. The modeling approach we used represents the US as a closed food system, such that all food demanded by consumers was produced domestically. We utilized this approach because US agencies provide high quality, publicly available data on the amount of agricultural resources used for individual crops, rather than for broad crop categories aggregated across diverse global regions. Additionally, the food loss and waste rates used in this study were sourced from LAFA, which may underestimate food loss and waste [71], and the limitations of this dataset have been noted elsewhere [72]. We recognize that food loss and waste may vary in ways not captured by our source data, but these are the most comprehensive, disaggregated, and contemporary data available at the national level. FCID was used as a crosswalk between LAFA and NHANES, but FCID has not been updated since 2010, and sensitivity analyses demonstrated that this approach underestimated daily per capita Total Food Demand by 181 g overall. No meaningful differences were observed across diet quality quintiles when these differences were expressed as a proportion of Total Food Demand mass, suggesting low likelihood of differential bias. FCID is uniquely able to disaggregate NHANES foods into sufficient resolution to be linked with individual LAFA commodities, which allows for an accounting of loss and waste for each NHANES food. Since food loss and waste represents 46% of Total Food Demand (766 g out of 1673 g), not accounting for this food would have substantially underestimated agricultural resource use. Nonetheless, efforts are needed to update FCID to link with contemporary NHANES surveys to reduce systematic bias in these analyses.

This study fills a research gap by focusing on the use of agricultural resources (notably fertilizer nutrients and pesticide use) rather than environmental impacts such as climate change [7]; yet the use of these resources is influenced by numerous factors that could not be incorporated into this analysis due to limited data availability, including the projected availability and intensity of agricultural inputs as a result of climate change [73]. The US Foodprint Model is a generalized instrument that utilizes national average data on biophysical processes, and therefore it does not explicitly incorporate characteristics of the US food system that vary spatially or by type of production system. For example, data on the application rates of agricultural amendments in certified organic operations is not part of the explicit model computations, but these data are nonetheless included in the national averages that the model uses. Additionally, the model does not estimate water scarcity, which can capture regional differences in water stress that are relevant to regional production of certain crops with high consumptive water footprints (e.g., fruits grown in the arid west). More work is needed by the federal government to create linkages between publicly available nutritional and agricultural databases, which can allow researchers to merge data on food intake with different production systems and spatial characteristics. Fortunately, efforts are underway at USDA to coordinate this transfer of nutritional and agricultural data through FoodData Central, which provides a foundation for data linkage and transfer [74], yet greater efforts are needed. Finally, measurement error cannot be ruled out when analyzing self-reported dietary data, since respondents may alter their reported food intake based on the perceived healthfulness of their food choices [75, 76]. However, self-reported dietary data provide a rich source of highly detailed information on the consumption of individual foods, and these data are necessary for estimating diet quality and comparing dietary patterns between groups [77].

## Conclusions

By integrating methods from nutritional epidemiology with food system science into an interdisciplinary modeling framework, this study reveals that the link between diet quality and environmental sustainability is more nuanced than previously understood. Higher diet quality was linked with greater Total Food Demand, retail loss, inedible portions, consumer waste, and consumed food. Higher diet quality was associated with lower use of agricultural land, but the relationship to fertilizer nutrients, pesticides, and irrigation water was dependent on the tool used to measure diet quality; this points to the influence that diet quality indices can have on the results of diet sustainability analyses and the need for standardized metrics. Over one-quarter of agricultural resources were used to produce edible food that was not consumed (retail loss and consumer waste). Urgent policy efforts are needed to achieve national and international goals for sustainable development and waste reduction, which include strong and unified leadership, greater investment in research and programming, and facilitated coordination across federal agencies [78]. In the meantime, consumers can make meaningful progress with practical tools they already have [79]. Our findings have important implications for the development of sustainable dietary guidelines, which requires balancing population-level nutritional needs with the environmental impacts of food choices.

## Supplementary information

**Additional file 1: Supplemental Figure 1.** Data sources, compilation, and output. LAFA, Loss-adjusted Food Availability data series; FCID, Food Commodity Intake Database; NHANES, National Health and Nutrition Examination Survey ^1^Includes retail loss, inedible portions, consumer waste, and consumed food. ^2^Meat and mixed meat dishes (beef and beef mixed dishes; pork and pork mixed dishes; poultry and poultry mixed dishes; seafood and seafood mixed dishes; meat sandwiches, burgers, sausages, and hotdogs; bacon; and other meat dishes) eggs and egg dishes; dairy (milk and cream, cheese); soup; grains and mixed grain dishes (bread; breakfast cereal; pancakes, waffles, and French toast; pastas and grain mixtures; pizza and calzones; and grain-based desserts); nuts and seeds; fruits and vegetables in mixed dishes (whole fruit and mixed fruit dishes; fruit/vegetable juice; dark green vegetables; yellow and orange vegetables; tomatoes and tomato mixtures; legumes; other vegetables); potatoes and potato mixed dishes; margarine, table oils, and salad dressings; salty snacks; Mexican dishes; other foods and dishes. ^3^Grains, fruits, vegetables, legumes, nuts, sweeteners, feed grains and oilseeds, hay, permanent pasture, and cropland pasture. ^4^Sum of nitrogen, phosphorus (P_2_O_5_), and potash (K_2_O). ^5^Sum of insecticides, herbicides, and fungicides.

**Additional file 2: Supplemental Figure 2.** Structure of the US Foodprint Model A comprehensive description of data sources, assumptions, supporting calculations, and structure of the US Foodprint Model can be found in Supplemental Text 1 in Peters et al. Carrying capacity of U.S. Agricultural land: Ten diet scenarios. *Elementa*. 2016;4. ^1^The model represents the US as a closed food system, such that all food demanded by consumers was produced domestically. Therefore, the demand for foods not produced domestically (bananas, coconuts, mangoes, pineapples, and some nuts) was proportionally apportioned to other foods in the same food group according to their availability in the USDA Loss-adjusted Food Availability data series. ^2^Conversion of plant-sourced foods from their “as consumed” form to their agricultural crop form, which adjusts for losses that occur before reaching the retail outlet (such as moisture loss and pre-retail waste). ^3^Conversion of animal-sourced from their “as consumed” form to their carcass weight, which adjusts for losses that occur before reaching the retail outlet (such as moisture loss and pre-retail waste, as well as bones and other non-marketable portions). Additional computations convert the carcass weight into livestock feed requirements by aligning livestock nutritional requirements with crop nutrient content; these computations are executed independently for each life phase for each livestock category (beef cattle, dairy cattle, swine, and poultry). ^4^Harvested product per acre. ^5^Adjustment for multiple food products produced from the same land parcel, to avoid double-counting acreage. ^6^Adjustment to account for unused grazing lands, to avoid overestimating the total amount of land currently grazed. Additional computations account for the portion of cropland that is used for grazing purposes. ^7^Restricts crop production and grazing to land that is agriculturally productive for each land-use type. For example, these computations prevent grazing land from being used as cropland.

**Additional file 3: Supplemental Table 1**. Scoring standards for each component of the Healthy Eating Index-2015 (HEI-2015) and Alternative Healthy Eating Index-2010 (AHEI-2010) EPA, eicosapentaenoic acid (20:5, n-3). DHA, docosahexaenoic acid (22:6, n-3). ^1^Development and validation available at https://epi.grants.cancer.gov/hei/developing.html#2005^2^Energy-adjusted to 1000 kcal, except for unsaturated fatty acids, saturated fatty acids, and added sugars. ^3^Cup equivalents and ounce equivalents were obtained from the USDA Food Pattern Equivalents Database (https://www.ars.usda.gov/northeast-area/beltsville-md-bhnrc/beltsville-human-nutrition-research-center/food-surveys-research-group/docs/fped-overview/). ^4^Development, validation, scoring standards, cup equivalents, and ounce equivalents from Chiuve et al. (2012). Alternative dietary indices both strongly predict risk of chronic disease. Journal of Nutrition, 142:1009–1018. To adapt the scoring standards for children, modifications were made to the scores for sodium and alcohol (see relevant footnotes for details), and the scores for the other components were energy-adjusted to 1849 kcal, which reflects the mean energy intake of the source population from which this index was constructed (from Chiuve et al., 2012). ^5^To adapt the scoring standards for children, scores were energy-adjusted to 1849 kcal, which reflects the mean energy intake of the source population from Chiuvre et al. (2012). Alternative dietary indices both strongly predict risk of chronic disease. Journal of Nutrition, 142:1009–1018. ^6^Beverages with ≥15 g of added sugar per 8 oz serving. ^7^Age categories (years): 1–3, 4–8, 9–13, 14–18, 19–30, 31–50, 51–70, ≥70. ^8^Adult non-drinkers received 2.5 points. Children (< 18 years) non-drinkers received 10 points and drinkers received 0 points.

**Additional file 4: Supplemental Table 2.** Characteristics of study population, National Health and Nutrition Examination Survey (2005–2016) ^1^Sample sizes are unweighted. ^2^Percentages within each column adjusted for survey weight.

**Additional file 5: Supplemental Table 3.** Healthy Eating Index-2015 component scores, National Health and Nutrition Examination Survey, 2005–2016 (*n* = 50,014) Includes individuals ≥2 years of age.

**Additional file 6: Supplemental Table 4.** Alternative Healthy Eating Index-2010 component scores, National Health and Nutrition Examination Survey, 2005–2016 (*n* = 50,014) EPA, eicosapentaenoic acid (20:5, n-3). DHA, docosahexaenoic acid (22:6, n-3). Includes individuals ≥2 years of age.

**Additional file 7: Supplemental Table 5.** Daily per capita Total Food Demand (2005–2016), by Healthy Eating Index-2015 quintile (*n* = 50,014) Higher quintiles represent higher diet quality. ^1^Test for linear trend across quintiles 1 through 5, not adjusted for covariates. ^2^Test for linear trend across quintiles 1 through 5, adjusted for age (continuous) and sex (male/female). ^3^Includes vegetable juice. ^4^Mostly candy, soft drinks, and other beverages.

**Additional file 8: Supplemental Table 6.** Daily per capita Total Food Demand (2005–2016), by Alternative Healthy Eating Index-2010 quintile (*n* = 50,014) Higher quintiles represent higher diet quality. ^1^Test for linear trend across quintiles 1 through 5, not adjusted for covariates. ^2^Test for linear trend across quintiles 1 through 5, adjusted for age (continuous) and sex (male/female). ^3^Includes vegetable juice. ^4^Mostly candy, soft drinks, and other beverages.

**Additional file 9: Supplemental Table 7.** Annual amount of agricultural land used to produce Total Food Demand, by land use category and Healthy Eating Index-2015 quintile Total Food Demand represents the sum of retail waste, consumer waste, inedible portions, and consumed food. Higher quintiles represent higher diet quality. HEI-2015, Healthy Eating Index-2015. ^1^Test for linear trend across quintiles 1 through 5.

**Additional file 10: Supplemental Table 8**. Annual amount of fertilizer nutrients used to produce Total Food Demand, by land use category and Healthy Eating Index-2015 quintile Total Food Demand represents the sum of retail waste, consumer waste, inedible portions, and consumed food. Higher quintiles represent higher diet quality. Fertilizers nutrients represents the sum of nitrogen, phosphorus (P2O5), and potash (K2O). HEI-2015, Health Eating Index-2015. NA, not applicable. ^1^Test for linear trend across quintiles 1 through 5.

**Additional file 11: Supplemental Table 9**. Annual amount of pesticides used to produce Total Food Demand, by land use category and Healthy Eating Index-2015 quintile Total Food Demand represents the sum of retail waste, consumer waste, inedible portions, and consumed food. Higher quintiles represent higher diet quality. Pesticides include herbicides, insecticides, and fungicides. HEI-2015, Health Eating Index-2015. NA, not applicable. ^1^Test for linear trend across quintiles 1 through 5.

**Additional file 12: Supplemental Table 10**. Annual amount of irrigation water used to produce Total Food Demand, by land use category and Healthy Eating Index-2015 quintile Total Food Demand represents the sum of retail waste, consumer waste, inedible portions, and consumed food. Higher quintiles represent higher diet quality. HEI-2015, Healthy Eating Index-2015. ^1^Test for linear trend across quintiles 1 through 5.

**Additional file 13: Supplemental Table 11.** Annual amount of agricultural land used to produce Total Food Demand, by Alternative Healthy Eating Index-2010 quintile Total Food Demand represents the sum of retail waste, consumer waste, inedible portions, and consumed food. Higher quintiles represent higher diet quality. AHEI-2010, Alternative Healthy Eating Index-2010. ^1^Test for linear trend across quintiles 1 through 5.

**Additional file 14: Supplemental Table 12**. Annual amount of fertilizer nutrients used to produce Total Food Demand, by Alternative Healthy Eating Index-2010 quintile Total Food Demand represents the sum of retail waste, consumer waste, inedible portions, and consumed food. Higher quintiles represent higher diet quality. Fertilizers include nitrogen, phosphorus (P2O5), and potash (K2O). AHEI-2010, Alternative Healthy Eating Index-2010. NA, not applicable. ^1^Test for linear trend across quintiles 1 through 5.

**Additional file 15: Supplemental Table 13**. Annual amount of pesticides used to produce Total Food Demand, by Alternative Healthy Eating Index-2010 quintile Total Food Demand represents the sum of retail waste, consumer waste, inedible portions, and consumed food. Higher quintiles represent higher diet quality. Pesticides include herbicides, insecticides, and fungicides. AHEI-2010, Health Eating Index-2010. NA, not applicable. ^1^Test for linear trend across quintiles 1 through 5.

**Additional file 16: Supplemental Table 14**. Annual amount of irrigation water used to produce Total Food Demand, by Healthy Eating Index-2010 quintile Total Food Demand represents the sum of retail waste, consumer waste, inedible portions, and consumed food. Higher quintiles represent higher diet quality. AHEI-2010, Alternative Healthy Eating Index-2010. ^1^Test for linear trend across quintiles 1 through 5.

**Additional file 17: Supplemental Table 15**. Daily per capita Total Food Demand by time period. HEI-2015, Healthy Eating Index. AHEI-2010, Alternative Healthy Eating Index. Total Food Demand includes retail loss, inedible, consumer waste, and consumption.. ^1^Z-tests with *P* < 0.05 tested the difference in diet quality scores within each quintile.

**Additional file 18: Supplemental Table 16.** Unadjusted and adjusted Alternative Healthy Eating Index-2010 scores (2005–2016). Adjusted scores reflect two changes to the Alternative Healthy Eating Index-2010 scoring algorithm to adapt this instrument to a population that includes individuals < 18 years: 1) consumption amounts were energy adjusted to the mean energy intake of the source population (1849 kcal/day), and 2) individuals < 18 years were awarded 10 points for the alcohol component if they reported zero consumption and were awarded zero points if they reported any alcohol consumption.. ^1^Wald tests with *P* < 0.05 tested the difference between unadjusted and adjusted scores within each age group.

## Data Availability

The datasets used in this study are publicly available: https://wwwn.cdc.gov/nchs/nhanes/Default.aspx;http://fcid.foodrisk.org/#;https://www.ers.usda.gov/data-products/food-availability-per-capita-data-system/

## Abbreviations

AHEI: Alternative Healthy Eating Index
DGA: Dietary Guidelines for Americans
EPA: Environmental Protection Agency
FCID: Food Commodity Intake Database
HEI: Healthy Eating Index
LAFA: Loss-adjusted Food Availability data series
NHANES: National Health and Nutrition Examination Survey
USDA: US Department of Agriculture
