# Supplementary figures and images for: Healthy diets can create environmental trade-offs, depending on how diet quality is measured
Source: Nutr J. 2020 Oct 27;19:117. doi: 10.1186/s12937-020-00629-6 (PMC7592508; doi:10.1186/s12937-020-00629-6)

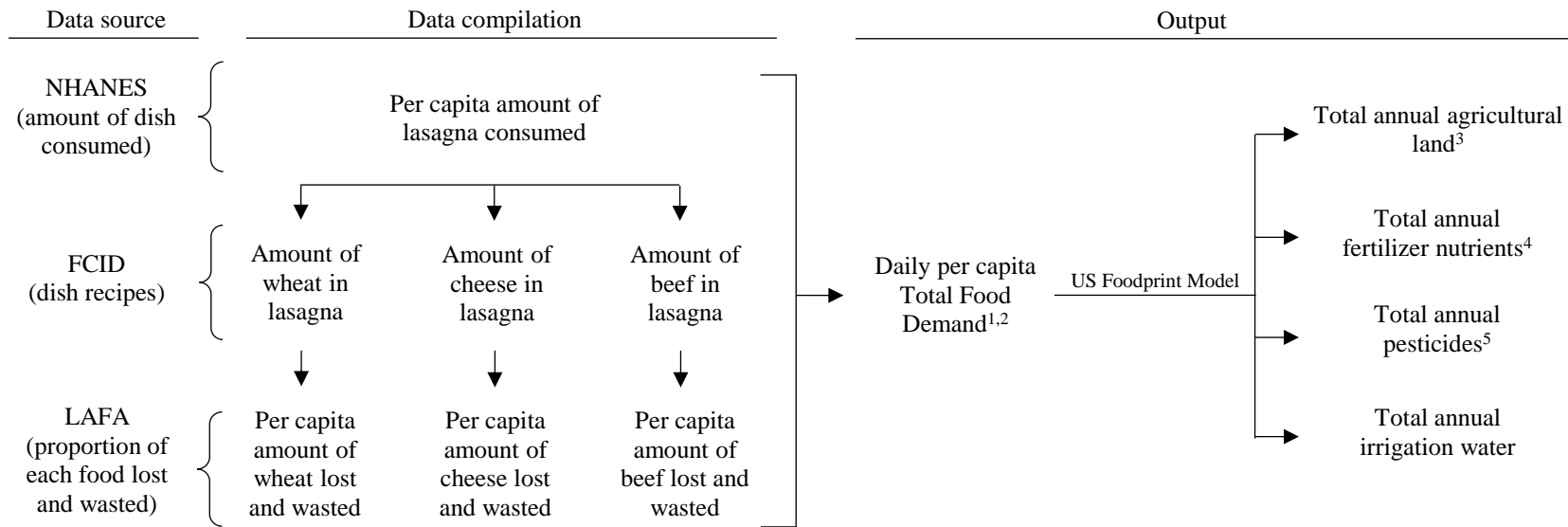

Supplement: Supplementary file 1 — Additional file 1: Supplemental Figure 1. Data sources, compilation, and output. LAFA, Loss-adjusted Food Availability data series; FCID, Food Commodity Intake Database; NHANES, National Health and Nutrition Examination Survey 1Includes retail loss, inedible portions, consumer waste, and consumed food. 2Meat and mixed meat dishes (beef and beef mixed dishes; pork and pork mixed dishes; poultry and poultry mixed dishes; seafood and seafood mixed dishes; meat sandwiches, burgers, sausages, and hotdogs; bacon; and other meat dishes) eggs and egg dishes; dairy (milk and cream, cheese); soup; grains and mixed grain dishes (bread; breakfast cereal; pancakes, waffles, and French toast; pastas and grain mixtures; pizza and calzones; and grain-based desserts); nuts and seeds; fruits and vegetables in mixed dishes (whole fruit and mixed fruit dishes; fruit/vegetable juice; dark green vegetables; yellow and orange vegetables; tomatoes and tomato mixtures; legumes; other vegetables); potatoes and potato mixed dishes; margarine, table oils, and salad dressings; salty snacks; Mexican dishes; other foods and dishes. 3Grains, fruits, vegetables, legumes, nuts, sweeteners, feed grains and oilseeds, hay, permanent pasture, and cropland pasture. 4Sum of nitrogen, phosphorus (P2O5), and potash (K2O). 5Sum of insecticides, herbicides, and fungicides. [file 12937_2020_629_MOESM1_ESM.pdf]

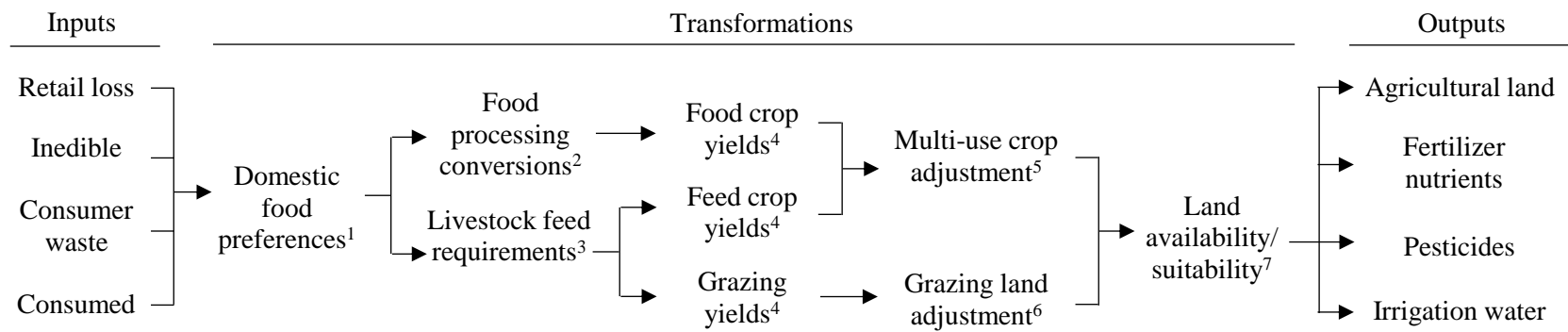

Supplement: Supplementary file 2 — Additional file 2: Supplemental Figure 2. Structure of the US Foodprint Model A comprehensive description of data sources, assumptions, supporting calculations, and structure of the US Foodprint Model can be found in Supplemental Text 1 in Peters et al. Carrying capacity of U.S. Agricultural land: Ten diet scenarios. Elementa. 2016;4. 1The model represents the US as a closed food system, such that all food demanded by consumers was produced domestically. Therefore, the demand for foods not produced domestically (bananas, coconuts, mangoes, pineapples, and some nuts) was proportionally apportioned to other foods in the same food group according to their availability in the USDA Loss-adjusted Food Availability data series. 2Conversion of plant-sourced foods from their “as consumed” form to their agricultural crop form, which adjusts for losses that occur before reaching the retail outlet (such as moisture loss and pre-retail waste). 3Conversion of animal-sourced from their “as consumed” form to their carcass weight, which adjusts for losses that occur before reaching the retail outlet (such as moisture loss and pre-retail waste, as well as bones and other non-marketable portions). Additional computations convert the carcass weight into livestock feed requirements by aligning livestock nutritional requirements with crop nutrient content; these computations are executed independently for each life phase for each livestock category (beef cattle, dairy cattle, swine, and poultry). 4Harvested product per acre. 5Adjustment for multiple food products produced from the same land parcel, to avoid double-counting acreage. 6Adjustment to account for unused grazing lands, to avoid overestimating the total amount of land currently grazed. Additional computations account for the portion of cropland that is used for grazing purposes. 7Restricts crop production and grazing to land that is agriculturally productive for each land-use type. For example, these computations prevent grazin [file 12937_2020_629_MOESM2_ESM.pdf]
